# Editorial: Genetic Regulation of Meat Quality Traits in Livestock Species

**DOI:** 10.3389/fgene.2022.1092562

**Published:** 2023-01-04

**Authors:** Rajwali Khan, Anning Li, Sayed Haidar Abbas Raza

**Affiliations:** ^1^ Department of Livestock Management, Breeding and Genetics, The University of Agriculture Peshawar-Pakistan, Peshawar, Pakistan; ^2^ College of Animal Science and Technology, Northwest A&F University, Yangling, China

**Keywords:** gene expression and regulation, meat quality, candidate gene, intramuscular fat (IMF), marker genes, differential gene expression

The increasing demand of the animal origin food for ever-expanding human population is an alarming call for the sustainable development in livestock productivity with high-quality animal proteins. In recent years, the application of modern technologies including advanced sequencing technologies, genome editing, genotype analysis, and genome profiling has promoted important changes in livestock genetic and breeding programs. These factors still fall short of accounting for the optimal level of variation that is required to achieve continued improvements in livestock productivity. The epigenome, which responds to internal and external environmental cues, is less explored but contains additional levels of variation that could be exploited for livestock traits of economic importance. Meat production and quality traits are strictly controlled by genetic factors, however their molecular mechanism is still needs to be explored. Intramuscular fat is a real challenge for the experts of animal science to improve meat quality traits. Research on the mechanism of adipogenesis provides invaluable information for the improvement of meat quality traits. In livestock species, the IM fat is considered one of the most important factors that determines carcass quality traits. Therefore, approaches for the improvement of Intramuscular fat deposition are crucial for the development of meat quality (Junjvlieke et al., 2020; Raza et al., 2020). Increasing the intramuscular fat content will increase the marbled texture of beef, and the tenderness and flavor of beef will become more palatable. Therefore, increasing the degree of fat deposition especially intramuscular fat in beef cattle is important research contents in the current beef cattle industry. Fat content is closely related to the number of fat cells and the volume of fat cells, and is regulated by the transcriptional expression of a large number of genes during the proliferation and differentiation of fat cells. At present, research on key regulatory genes in adipocyte differentiation and lipid metabolism is a focus of attention (Guo et al., 2018a; Guo et al., 2018b; Hongfang et al., 2022). Therefore, the current Research Topic was planned and researchers around the globe were invited to explore the mechanism of genetic regulation of meat quality traits in livestock species. All important molecular mechanisms such as transcriptional and post-transcriptional regulation of adipogenic marker genes. RNAs, transcription factors, candidate genes, SNPs, sequencing, DNA methylation, DNA-Protein interaction for the improvement of meat quality traits were considered in this Research Topic. The Research Topic was published in 24 February 2022 with the titled “*Genetics regulation of meat quality traits in livestock specie*s”, and closed on 31st of August 2022. A total of 09 manuscripts were published by 73 authors around the globe. Keeping in view the importance of molecular regulation of meat quality traits of meat obtained from different livestock species, we received manuscripts regarding cattle, buffalo, yak, sheep, pig and donkeys. We can summarize the published articles with the following description against each group of manuscript based on the target species of livestock.

In the first category, the manuscript related with beef quality from cattle were published in this Research Topic. The first manuscript explored the role of *N* ^6^-methyladenosine (m^6^A) in bovine skeletal myoblast differentiation. They ascertained mRNA m^6^A methylation exhibited declined changes during bovine skeletal myoblast differentiation, and both *MEF2C* mRNA expression and m^6^A levels were significantly increased during myoblast differentiation. They also found that *MEF2C* with mutated m^6^A sites significantly inhibited myoblast differentiation compared with wild-type *MEF2C*. METTL3 promoted MEF2C protein expression through posttranscriptional modification in an m^6^A-YTHDF1-dependent manner. Moreover, MEF2C promoted the expression of METTL3 by binding to its promoter. These results revealed that there is a positive feedback loop between these molecules in myoblast differentiation. This manuscript provided new insights into skeletal muscle differentiation and fusion, which may provide an RNA methylation-based approach for molecular genetics and breeding in livestock as well as for the treatment of muscle-related diseases (Yang et al.). The second manuscript used bioinformatics tools for the exploration of differential genes in the regulation of adipogenesis. The authors downloaded the GSE116775 microarray dataset from Gene Expression Omnibus (GEO). The weighted gene co-expression network (WGCNA) was used to analyze the gene expression profile, and the key gene modules with the highest correlation with subcutaneous adipose tissue were identified, and the functional enrichment of the key modules was analyzed. Then, the “real” Hub gene was screened by in-module analysis and protein–protein interaction network (PPI), and its expression level in tissue samples and adipocytes was verified. The study showed that a total of nine co-expression modules were identified, and the number of genes in these modules ranged from 101 to 1,509. Among them, the blue module is most closely related to subcutaneous adipose tissue, containing 1,387 genes. These genes were significantly enriched in 10 gene ontologies including extracellular matrix organization, biological adhesion, and collagen metabolic process, and were mainly involved in pathways including ECM-receptor interaction, focal adhesion, cAMP signaling pathway, PI3K-AKT signaling pathway, and regulation of lipolysis in adipocytes. In the PPI network and coexpression network, five genes (CAV1, ITGA5, COL5A1, ABL1, and HSPG2) were identified as “real” Hub genes. Analysis of Hub gene expression by dataset revealed that the expression of these Hub genes was significantly higher in subcutaneous adipose tissue than in other tissues. In addition, real-time fluorescence quantitative PCR (qRT-PCR) analysis based on tissue samples and adipocytes also confirmed the above results. In this study, five key genes related to subcutaneous adipose tissue were discovered, which laid a foundation for further study of the molecular regulation mechanism of subcutaneous adipose tissue development and adipose deposition (Sheng et al.).

In this Research Topic two manuscripts were published regarding exploration of molecular mechanism in buffalo meat quality. In the first manuscript, the authors explore the role of Fatty acid transport 1 (FATP1) in intramuscular adipogenesis in buffalo. They investigated the effects of FATP1 on the differentiation and proliferation of adipocytes in five types of cells derived from muscle and adipose tissue and estimated the effects of FATP1 on intramuscular fat (IMF) deposition. This manuscript explored that FATP1 is mainly expressed in heart and muscle tissue in buffaloes as well as cells undergoing adipogenic differentiation. Interestingly, they found that FATP1 promoted the adipogenic differentiation of muscle-derived cells (buffalo myocytes and intramuscular preadipocytes and mouse C2C12 cells) but did not affect, or even inhibited, that of adipose-derived cells (buffalo subcutaneous preadipocytes and mouse 3T3-L1 cells, respectively). They concluded that FATP1 promotes IMF deposition and enhance the proliferative activity of all the assessed cells, except murine 3T3-L1 cells. These results provide new insights into the potential effects of FATP1 on IMF deposition, especially regarding its positive effects on meat quality in buffaloes and other livestock species (Huanget al.). In the second manuscript regarding buffalo meat quality, the authors used the bioinformatics tools along with the *in-vivo* and *in-vitro* analysis for the exploration of molecular mechanism in buffalo meat. In this study, weighted gene co-expression network analysis (WGCNA) was used to construct a circRNA co-expression network and revealed a candidate circRNA that may affect the IMF deposition of buffalo as determined by RT-qPCR, semiquantitative PCR and gain-of-function experiments. In this manuscript, the WGCNA determined that one module (turquoise module) is significantly associated with the growth and development stages of buffalo. Further analysis revealed a total of 191 overlapping circRNAs among differentially expressed (DE) circRNAs and the co-expression module. A candidate circRNA was found, 21:6969877|69753491 (circRNA_ID), with a reported involvement in lipid metabolism. This circRNA is stably expressed and originates from the *MARK*3 gene, hence the name circMARK3. The circMARK3 is highly expressed in adipose tissue and mature adipocytes and is located in the cytoplasm. Gain-of-function experiments demonstrated that circMARK3 promoted adipogenic differentiation of buffalo adipocytes and 3T3-L1 cells by up-regulating the expression levels of adipogenic marker genes *PPARG, C/EBP*α and *FABP*4. They concluded that circMARK3 is a potential factor that promotes fat deposition by regulating adipocyte differentiation and adipogenesis in buffalo (Feng et al.).

In the third group of manuscripts, the articles related to donkey meat quality were included. One manuscript published in this category explored a total of 585,555 spectra from the six muscle samples. In total, 20,583 peptides were detected, including 15,279 unique peptides, and 2,540 proteins were identified. They, analyzed differentially abundant proteins (DAPs) between longissimus dorssi (LD) muscles of donkeys with high (H) and low (L) IMF content. They identified 30 DAPs between the H and L IMF content groups, of which 17 were upregulated and 13 downregulated in the H IMF group. Gene Ontology (GO) and Kyoto Encyclopedia of Genes and Genomes (KEGG) functional enrichment analysis of these DAPs revealed many GO terms (e.g., bone morphogenetic protein (BMP) receptor binding) and pathways (e.g., Wnt signaling pathway and Hippo signaling pathway) involved in lipid metabolism and adipogenesis. The construction of protein–protein interaction networks identified 16 DAPs involved in these networks. Our data provide a basis for future investigations into candidate proteins involved in IMF deposition and potential new approaches to improve meat quality in the donkey (Tan et al.).

The fourth group of published articles in this Research Topic included molecular regulation of mutton quality traits obtained from sheep. In one manuscript the authors characterized the miRNA transcriptome of the longissimus thoracis et lumborum muscle at several developmental time points such as at gestational d 85 (PN1), 110 (PN2), 133 (PN3), postnatal d 42 (PW1), 65 (PW2), and 243 (MAT) during muscle hypertrophy in lambs. They also examined the miR-29a expression and found that it is differentially regulated across development, loss of function on satellite cell proliferation and differentiation. Muscle fiber characteristics showed drastic increases (*p* < 0.0001) in fiber size and alterations in muscle fiber type occur during pre and postnatal development. miRNA sequencing comparisons were performed in developmental order (PN1 vs. PN2, PN2 vs. PN3, PN3 vs. PW1, PW1 vs. PW2, PW2 vs. MAT). There were 184 differentially expressed (Padj <0.05) miRNA, 142 unique miRNA, from all 5 comparisons made. The transitional stage (PN3 vs. PW1) had the largest number (115) of differentially expressed miRNA. Inhibition of miR-29a in satellite cell culture increased (*p* < 0.05) cell proliferation and differentiation capacity. Characterization of the miRNA transcriptome provides valuable insights into the miRNA involved in muscle fiber hypertrophy and the potential importance of the transitional period (Greene et al.). In another manuscript, in this category of sheep mutton quality the authors used high-throughput RNA sequencing to assess the expression profiles of coding and non-coding RNAs in muscle tissue of Tan sheep and Dorper sheep. To investigate the molecular processes involved in the formation of muscle fibers, they collected two different muscle tissues, longissimus dorsi and biceps femoris, from Tan sheep and Dorper sheep. The longissimus dorsi of Tan sheep and Dorper sheep displayed significantly differential expression levels for 214 lncRNAs, 25 mRNAs, 4 miRNAs, and 91 circRNAs. Similarly, 172 lncRNAs, 35 mRNAs, 12 miRNAs, and 95 circRNAs were differentially expressed in the biceps femoris of Tan sheep and Dorper sheep according to the expression profiling. GO and KEGG annotation revealed that these differentially expressed genes and non-coding RNAs were related to pathways of the formation of muscle fiber, such as the Ca^2+^, FoxO, and AMPK signaling pathways. Several key genes are involved in the formation of muscle fibers, including ACACB, ATP6V0A1, ASAH1, EFHB, MYL3, C1QTNF7, SFSWAP, and FBXL5. RT-qPCR verified that the expression patterns of randomly selected differentially expressed transcripts were highly consistent with those obtained by RNA sequencing. A total of 10 lncRNAs, 12 miRNAs, 20 circRNAs, and 19 genes formed lncRNA/circRNA-miRNA-gene networks, indicating that the formation of muscle fiber in Tan sheep is controlled by intricate regulatory networks of coding and non-coding genes. Our findings suggested that specific ceRNA subnetworks, such as circ_0017336-miR-23a-FBXL5, may be critical in the regulation of the development of muscle fibers, offering a valuable resource for future study of the development of muscle fibers in this animal species. These findings increase our understanding of the variety in how muscle fibers originate in various domestic animals and lay the groundwork for future research into new systems that regulate the development of muscle (Cui et al.).

In this Research Topic the fifth category of published articles was regarding the exploration of molecular mechanism of pig meat quality. The first article the circRNA expression profiles between Huainan pigs (Chinese indigenous pigs, fat-type, HN) and Large White pigs (Western commercial pigs, lean-type, LW) in the longissimus dorsi (LD) muscle at 38, 58, and 78 days post conception (dpc) were compared by sequencing. In total, 39,887 circRNAs were identified in 18 samples, and 60, 78, and 86 differentially expressed circRNAs (DECs) were found at the three stages mentioned above between these two breeds. The parent genes of DECs were enriched in myogenesis, proliferation, adipogenesis and muscle fiber-type transition. The circRNA-miRNA interaction networks included 38 DECs and 47 miRNAs, and these miRNAs were involved in muscle development and lipid metabolism. Two shared DECs (circ_0030593 and circ_0032760) of these three stages were selected, their head-to-tail junction sites were validated by Sanger sequencing, and RT‒qPCR results suggested that these two DECs might be involved in intramuscular fat deposition. These findings provide a basis for understanding the role of circRNAs in meat quality (Wang et al.). In the second manuscript under this category, the authors analyzed lncRNAs in longissimus thoracis (LT) and semitendinosus (ST) muscles, being different in meat quality, with RNA-sequencing technology. A total of 500 differentially expressed lncRNAs (DELs) and 2,094 protein-coding genes (DEGs) were identified. Through KEGG analysis on DELs, they first made clear that fat deposition might be the main reason resulting in the differential phenotype of LT and ST, for which cGMP–PKG and VEGF signaling pathways were the most important ones. In total, forty-one key DELs and 50 DEGs involved in the differential fat deposition were then characterized. One of the key genes, cAMP-response element binding protein 1, was selected to confirm its role in porcine adipogenesis with molecular biology methods and found that it promotes the differentiation of porcine preadipocytes, consistent with its higher expression level and intramuscular fat contents in LT than that in ST muscle. Furthermore, through integrated analysis of DELs and DEGs, transcription factors important for differential fat deposition were characterized among which BCL6 has the most target DEGs while MEF2A was targeted by the most DELs. The results provide candidate genes crucial for meat quality, which will contribute to improving meat quality with molecular-breeding strategies (Sun et al.).
